# A survey of oral surgeons’ tobacco-use-related knowledge and intervention behaviors

**DOI:** 10.4317/medoral.17724

**Published:** 2012-02-09

**Authors:** Raquel González-Martínez, Esther Delgado-Molina, Cosme Gay-Escoda

**Affiliations:** 1 DDS. Resident of the Master of Oral Surgery and Implantology. University of Barcelona Dental School; 2 MD, DDS. Master in Oral Surgery and Implantology. Professor of the Master of Oral Surgery and Implantology. University of Barcelona Dental School. IDIBELL Research group; 3 MD, DDS, PhD. Chairman and professor of Oral and Maxillofacial Surgery. Director of the Master in Oral Surgery and Implantology. University of Barcelona Dental School. Oral and maxillofacial surgeon of the Teknon Medical Center, Barcelona (Spain). IDIBELL Research group

## Abstract

Objectives: To evaluate whether oral surgeons are aware of tobacco’s role in oral health. Moreover, we wanted to know professionals’ attitudes towards smoker patients and physicians’ involvement in detecting and eradicating this habit in patients. Study Design: We conducted a survey to determine the awareness of the members of the Spanish Society of Oral Surgery about tobacco’s damage on oral health and the role of dentists in the prevention and elimination of the smoking habit. Results: 450 surveys were distributed during the Seventh National Congress of the Spanish Society of Oral Surgery, of which 224 (49.8%) were answered. Seventy-six point eight percent of oral surgeons said that they have a good knowledge of the effects of snuff on oral health. However, only 42.9% admitted they had received specific training regarding how to deal with patients who want to give up smoking. Sixty-three point four percent had explained to smoker patients the risk of this habit for the oral and general health. However, 17% admitted they do not advise their patients to give up smoking for fear of upsetting them, while 15.2% expressed lack of time, and 3.6% think it is not their competence. As to the relationship between oral cancer and smoking, 83% of oral surgeons recognize a direct relationship. In addition, 85.7% of professionals believe that dentists have a primary role in oral cancer prevention. Conclusions: These results indicate that most oral surgeons are concerned about the smoking habit of their patients. However, it is necessary to increase the specific training of dentists by providing tobacco treatment programs as part of their professional responsibility. Oral surgeons recognize the direct relationship between the smoking habit and oral cancer and regard as very important the role of dentists in the prevention of this disease.

** Key words:**Smoking habit, oral surgery, oral cancer.

## Introduction

Tobacco use is one of the most important causes of oral cancer and periodontal disease. There is a direct relationship between tobacco use and these oral pathologies. Also, it has been observed that smoker patients have a higher incidence of oral lesions such a leukoplasia, which disappears with smoking cessation, and other alterations in the oral mucosa, such as nicotine stomatitis, gingival recessions, acute necrotizing ulcerative gingivitis, calculus formation and halitosis (1).

Smoking has killed more than 60,000 people in Spain over the last year and has become the leading cause of illness, disability and avoidable death. In addition; 25% of deaths attributed to tobacco use occur prematurely. However, despite the risk posed by tobacco dependence for general health, the prevalence of smoking in the Spanish population older than 15 years was 34.4% in 2001 (2).

Between 10% and 20% of smokers patients who have received professional support and appropriate assistance from physicians have succeeded in smoking cessation (3-5).

Dental practice is the perfect scenario to apply prevention treatments, oral screening and the oral health education of patients. The majority of the population sees a dentist more regularly than any other health professionals, especially in certain age groups. Over 60% of adults and 83% of the population between 15 and 19 years of age see a dentist at least once in a year. This interaction allows dentists to actively work in the cessation and eradication of tobacco dependence in the population (1).

This article discusses the involvement of dentists in the prevention and eradication of tobacco. The attitude of oral health professional with tobacco users is also evaluated.

## Material and Methods

A cross-sectional study was carried out by distributing 450 polls to members of the Spanish Society of Oral Surgery who attended to the Seventh National Congress of this Society in Almería (Spain) in October 2009.

The members of the scientific society – chosen at random – were asked about their implication in tobacco dependence. The only requirement to participate in the study was to be a member of the Spanish Society of Oral Surgery.

The questionnaire was anonymous. In an attempt to find patterns of behavior by groups, participants were requested to indicate their gender, age and personal data, and years of professional practice. The survey was a 21-item multiple choice. It was about seven topics: 1) professionals’ knowledge on the relationship between smoking and oral health, 2) their interest in identifying patients who smoke, 3) their involvement to help smokers give up the habit, 4) the training they had received on tobacco dependence, and the impact the habit has on oral and general health, as well as how to treat smokers, 5) the relationship between tobacco use and oral cancer, 6) the relationship between smoking and postoperative complications and 7) the impact of tobacco use on periodontal health.

The questionnaire had a time limit, but all questions could be answered in a few minutes. All surveys collected were included in the study, even though not all sections were answered.

Data were analyzed using the Statistical Package for Social Science version 15.0 for Windows (SPSS inc., Chicago, IL, USA. License granted to the University of Barcelona).

## Results

Four hundred and fifty polls were distributed, of which 224 were completed, and represented 49.77% of participation. Ninety-four percent of respondents were men (41.9%) and 130 women (58%). The mean age was between 23 and 65 years, and the average time of professional practice ranged from 1 to 37 years. There were no statistically significant differences between gender, age or years of professional practice in none of the questions.

**Figure 1:**
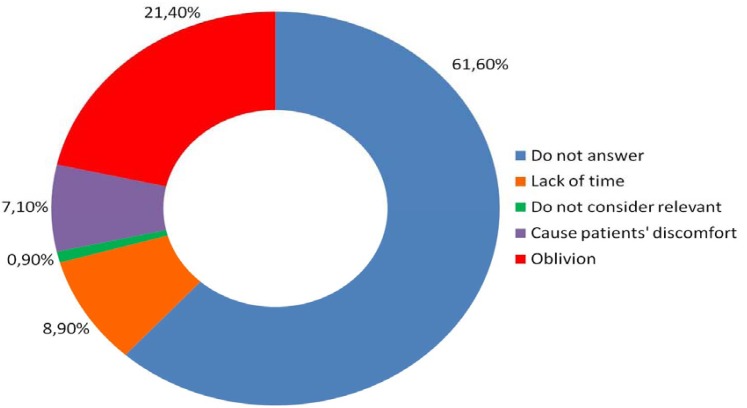
Reasons why professionals surveyed do not ask their patients about tobacco dependence during the first visit.

Seventy-six point eight percent of oral surgeons indicated they had a comprehensive knowledge of the effects of tobacco use on oral health. However, only 42.9% said they had received professional training regarding how to deal with the tobacco dependence in patients who want to give up the habit. Despite the lack of training, 75.9% say they always ask their patients about tobacco use during the first visit. The most mentioned reason for avoiding this question is they forget about it during the visit (21.4%), and 8.9% affirmed they had not much time to discuss it with their patients (Fig. 1).

**Figure 2:**
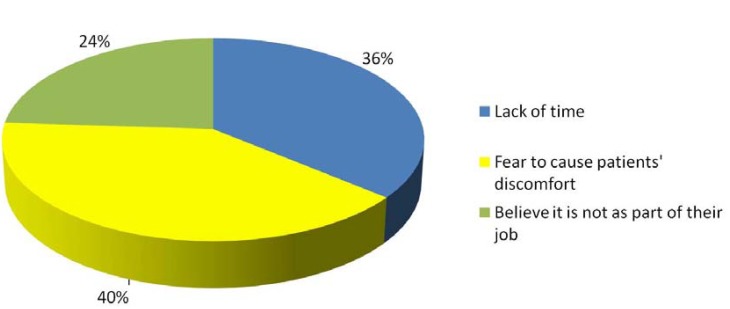
Reasons why professionals surveyed do not advice smoker patients to give up smoking.

Sixty-three point four percent answered that they explained to smoker patients the risks of tobacco use and its effects on oral and general health, while and 67.9% of dentists advised to give up the habit. Professionals who do not involve in their patients’ tobacco dependence indicated the following reasons: fear of upsetting their patients (40.47%); lack of time (36.19%) and the belief that tobacco treatment is not their professional responsibility (23.57%) (Fig. 2). Regarding the relationship between oral cancer and tobacco use, 83% of oral surgeons recognize a direct relationship. In addition, 85.7% of professionals surveyed believe the primary role of dentists in oral cancer is prevention.

As to the relationship between tobacco use and oral surgery, most dentists ask their patients how many cigarettes they usually smoke every day. In addition, 34.8% of professionals consider essential to cease the habit before implant surgery. Eighty-eight point four percent consider tobacco dependence as a key factor in the failure of implants, mainly when consumption is more than 10 cigarettes a day.

With reference to the impact of tobacco use on periodontal health, 85.7% of professionals say there is a direct and widely known relationship between smoking and periodontal disease.

**Figure 3:**
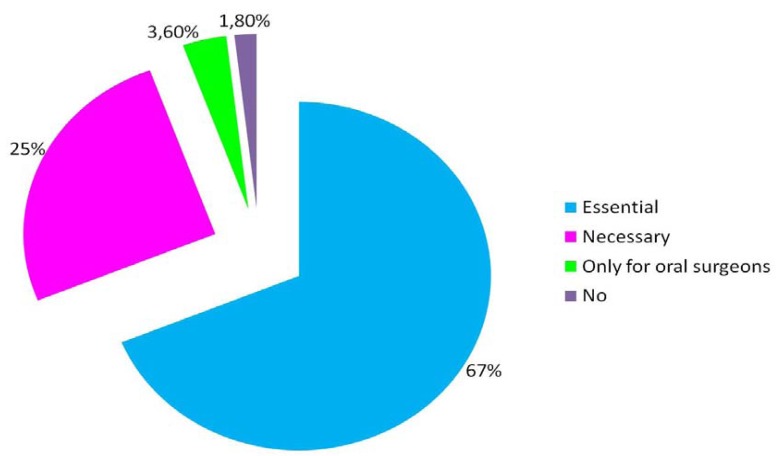
How important is for those professionals surveyed the incorporation of continuous specific training courses to learn how stop tobacco use.

Finally, 67% of participants believe it is essential to include a continuous training of all health professionals for the management and discontinuation of tobacco use (Fig. 3).

## Discussion

The leading causes of death attributed to tobacco dependence are as follows: lung cancer (26.5%), chronic obstructive pulmonary disease (20.9%), ischemic heart disease (12.8%) and cardiovascular disease (9.2%) (2).

According to the latest data released by the Ministry of Health of Spain, tobacco use has killed more than 60,000 people over the last year and has become the leading cause of illness, disability and avoidable death in this country. When tobacco dependence in Spain was analyzed during the period 1987-2001, it showed a reduction in tobacco use among men that varied from 55% to 42.1%. However, there was an increase in women’s smoking (from 23% to 27.2%). This increase is particularly significant among women aged 16 to 24 years, whose prevalence is 42.7% higher than men of that age group (40.8%). The age of smoking initiation is 13.1 years, with an average daily consumption of 7.4 cigarettes. The most worrisome fact is that the age of smoking initiation is increasingly lower, which makes necessary to redouble efforts in prevention policies aimed at adolescents (2).

The incidence of mortality is especially virulent for men. According to a study developed by the Spanish Ministry of Health in 1998, 92.5% of deaths from tobacco use occurred in men and only 7.5% in women. However, these data may be soon changing because more women have started to smoke in the last twenty years, with the subsequent increase in tobacco-related diseases and deaths among smoker women. In fact, and according to data published in 2000, lung cancer has undergone a 22% increase in women versus 2% in men in the last decade (2).

The prevalence of tobacco dependence in the Spanish population older than 15 years was 34.4% in 2001. These figures have dropped in comparison with those from 1987, when the percentage was 38.4%. Recently, smoking cessation rates in patients who have received professional support and appropriate assistance from their doctors have varied from 10% to 20% (2).

The majority of oral health professionals surveyed (94.7%) reported their concern for the smoking habits of their patients, advised them and tried to help them in giving up the habit. However, only 22.3% of the professionals admitted having received training courses in the management of patients’ tobacco dependence. In fact, there is not any specific formation in dentistry courses offered by the Spanish universities to train professionals to actively eradicate tobacco consumption.

The U.S. Public Health Service (PHS) Clinical Practice Guidelines recommends health care providers to brief patients on the risks of tobacco during their visits to the doctor’s office (6-12). In 2008, the PHS updated the Clinical Practice Guideline to be used by dentists. The guideline recommends the use of the “5 A’s”: 1) “Ask”: ask every patient about tobacco use at every visit, 2) “Advice”: advise every tobacco user to quit, 3) “Assess”: assess interest and confidence in quitting tobacco use, 4) “Assist”: assist interested tobacco users by setting a quit date and providing counseling and medication, 5) “Arrange”: arrange for timely follow-up services (6). According to Crews et al. (6) advices alone result in a small but reliable increase in patient cessation rates, while consistent performance often achieves better results. Despite these PHS recommendations, a study conducted by Kast et al. (13) in the state of Colorado reported that only 9% of adolescents have consulted their dentists about tobacco habit.

Surveys conducted among British and Canadian dentists by Stacey et al. (14) and Warnakulasuriya et al. (15), respectively, reported that most professionals claimed to be involved in the eradication of the smoking habit, a result that is consistent with our study of Spanish dentists. However, Stacey et al. (14) and Campbell et al. (1) found that the main reason by which British dentists did not become involved in their patients’ tobacco cessation was the lack of training. British professionals report a lack of knowledge to know how to help smoker patients (16-18). In our study, fear of causing patient discomfort (40%) and lack of time (37%) were the most mentioned reasons.

As with 22.3% of Spanish surgeons surveyed in our study, 20.2% of Americans admit that they have not received specific training on how to act with smoker patients. Seventy-one point four percent of American professionals believe it is essential to incorporate additional training courses for oral health professionals (6). We have observed that 67% of Spanish surgeons also demand this training.

Regarding the relationship between tobacco dependence and oral cancer, Warnakulasuriya et al. (15) reported that 95% of British dentists suspected the presence of oral cancer when making differential diagnoses of soft tissue injury in the oral cavity of smokers. Seventy four percent of these professionals usually refer the patient to a hospital, compared with 21% who do the biopsy in their professional practice. In Spain, 83% of oral surgeons assume a direct relationship between tobacco use and oral cancer.

There is a clear link between tobacco habit and periodontal disease (19-20). On the other hand, the presence of bacterial plaque and peri-implant bone loss was significantly higher in smokers (21). Vehemente et al. (22) found a risk of implant failure 4.3 times greater in smoker patients. Among the oral surgeons surveyed in our study, 34.8% consider essential to quit smoking before implant surgery, while 62.5% of them take into account the number of cigarettes and therefore recommend reducing the habit.

In conclusion, these findings indicate that there is a similarity of data with other countries; the members of the Spanish Society of Oral Surgery are consequently concerned about their patients’ tobacco use. In addition, they are worried about the relationship between the tobacco use and oral cancer. They are identifying tobacco users at a greater frequency than many other dental providers. Due to the lack of specific training, we consider essential to provide training experiences for dentists; it is crucial to achieve a continued progress in reducing the prevalence of tobacco-related diseases in our society.
